# Supplementary material for: High-temperature flexible WSe2 photodetectors with ultrahigh photoresponsivity
Source: Nat Commun. 2022 Jul 28;13:4372. doi: 10.1038/s41467-022-32062-0 (PMC9334605; doi:10.1038/s41467-022-32062-0)
Supplement: Supplementary file 1 — Supplementary Information [file 41467_2022_32062_MOESM1_ESM.pdf]

High-Temperature Flexible WSe_2 Photodetectors with Ultrahigh Photoresponsivity

Yixuan Zou[1,2,3], Zekun Zhang[1,2,3], Jiawen Yan[1,2,3], Linhan Lin[1], Guanyao Huang[4], Yidong Tan*[1], Zheng You*[1,2,3], Peng Li*[1,2,3]

[1]State Key Laboratory of Precision Measurement Technology and Instruments, Department of Precision Instruments, Tsinghua University, Beijing 100084, China.
[2]Key Laboratory of Smart Microsystem (Tsinghua University) Ministry of Education, Beijing 100084, China.
[3]Beijing Advanced Innovation Center for Integrated Circuits, Beijing 100084, China.
[4]Key Laboratory for Thermal Science and Power Engineering of Ministry of Education, Beijing Key Laboratory of CO_2 Utilization and Reduction Technology, Department of Energy and Power Engineering, Tsinghua University, Beijing 100084, China.

Supplementary Figures

Supplementary Figure 1. Optical images of step-by-step transfer process of the stacked van der Waals heterostructures.

Supplementary Figure 2. Atomic force microscope (AFM) topographic image and height-position curve of h-BN.

Supplementary Figure 3. Raman spectra of h-BN and Graphite flake.

Supplementary Figure 4. High Resolution Transmission electron microscope (HRTEM) images of the stacked structure and elemental analysis results of different areas.

Supplementary Figure 5. Optical images of WSe_2 FET before and after 700 °C/750 °C burning.

Supplementary Figure 6. Thermal stability of bare WSe_2 and WSe_2 covered with 100 nm Al_2O_3.

Supplementary Figure 7. WSe_2 FET before and after 1000 °C burning in vacuum.

Supplementary Figure 8. Dark current before and after 700 °C in air and 1000 °C in vacuum annealing.

Supplementary Figure 9. Optical microscope image of a typical device with Pt electrodes before and after annealing.

Supplementary Figure 10. Atomic force microscope (AFM) images of HOPG (highly oriented pyrolytic graphite), GF, and Pt electrode.

Supplementary Figure 11. Comparison of GF and Pt as bottom encapsulation layer.

Supplementary Figure 12. Photoluminescence spectroscopy image of WSe_2 in heat resistant structure from 150 °C to 500 °C.

Supplementary Figure 13. I_{ds}-V_{gs} curves (V_{ds}=0.8V) measured at room temperature before and after burning 15 min at 500 °C in air.

Supplementary Figure 14. Conducting properties of GF electrode at high-temperature.

Supplementary Figure 15. Photo responsivity of WSe_2 device with mica encapsulation under 385 nm (purple), 440 nm (blue), and 520 nm (green) illumination at different temperatures (V_{ds}=0.4 V, V_g=0 V).

Supplementary Figure 16. The I_{ds}-V_{ds} curves of bare WSe_2 with GF electrodes (without top h-BN) under different light intensity and temperature.

Supplementary Figure 17. The I_{ds}-V_{ds} curves of bare WSe_2 with Pt electrodes (without top h-BN) under different light intensity and temperature.

Supplementary Figure 18. The I_{ds}-V_{ds} curves of WSe_2 with Pt S/D electrodes and h-BN encapsulation (without top gate) under different light intensity and temperature.

Supplementary Figure 19. Schematic of transfer curves at different temperatures.

Supplementary Figure 20. Negative photoconductivity phenomenon at 400 ℃ and 500 ℃ under 0.2 W/m^2 365 nm light illumination.

Supplementary Figure 21. Noise current sequence at different temperatures.

Supplementary Tables

Supplementary Table 1. Devices with different sizes of top h-BN

Supplementary Table 2. Signal-to-noise ratio of the device at different temperatures

Supplementary Table 3. Comparation of representative photodetectors

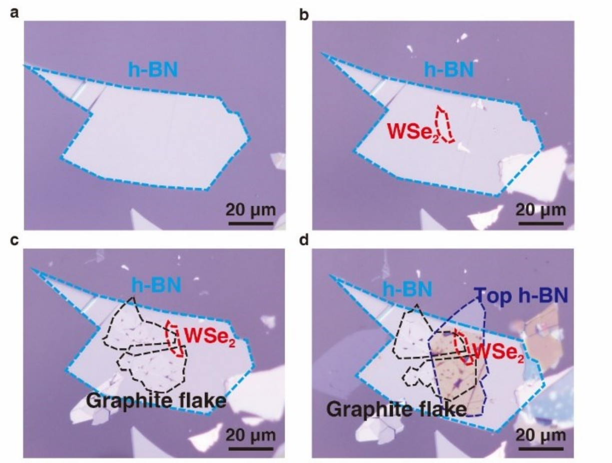

Supplementary Figure 1. Optical images of step-by-step transfer process of the stacked van der Waals heterostructures. a, Transfer of the bottom h-BN. **b,** Transfer of the WSe_2. **c,** Transfer of the two GF electrodes. **d,** Transfer of the top h-BN. Dashed lines are used to indicate the outline of WSe_2 (red), graphite flake (black), bottom h-BN (light blue) and top h-BN (dark blue).

Note: We exfoliated the mica sheet to about 100 μm thick to obtain clean, flat and flexible substrate. We cleaned the substrate using an ultrasonic cleaner to remove possible residues from the substrate surface. Then, mechanically exfoliated h-BN, WSe_2 and two pieces of GF as electrodes were transferred to the substrate step by step, as shown in Supplementary Figure 1a, 1b, 1c. Another h-BN was transferred to form fully encapsulation, as shown in Supplementary Figure 1d.

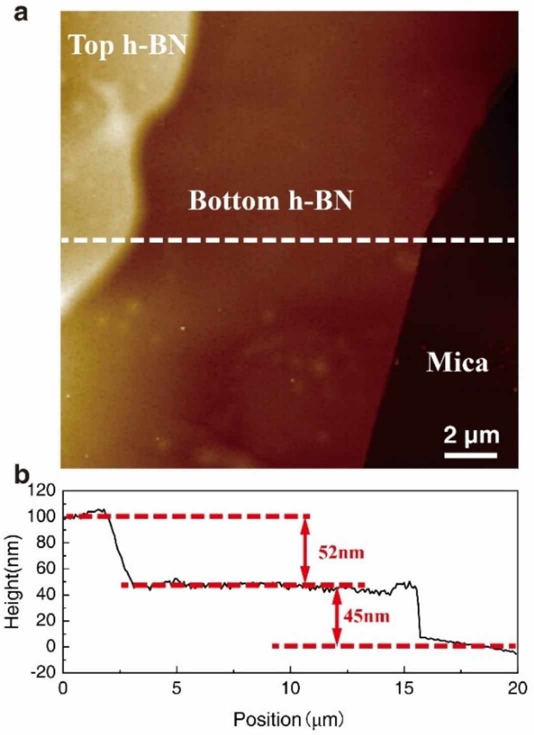

Supplementary Figure 2. Atomic force microscope (AFM) topographic image and

height-position curve of h-BN. a, AFM topographic image of bottom and top h-BN. **b,** Height-position curve along the dotted line in Supplementary Figure 2a. The thickness of top h-BN is 52 nm and the bottom h-BN is 45 nm. Generally, 40-60 nm thick h-BN were preferred to be chosen considering both the thermal protection and gate dielectric isolation.

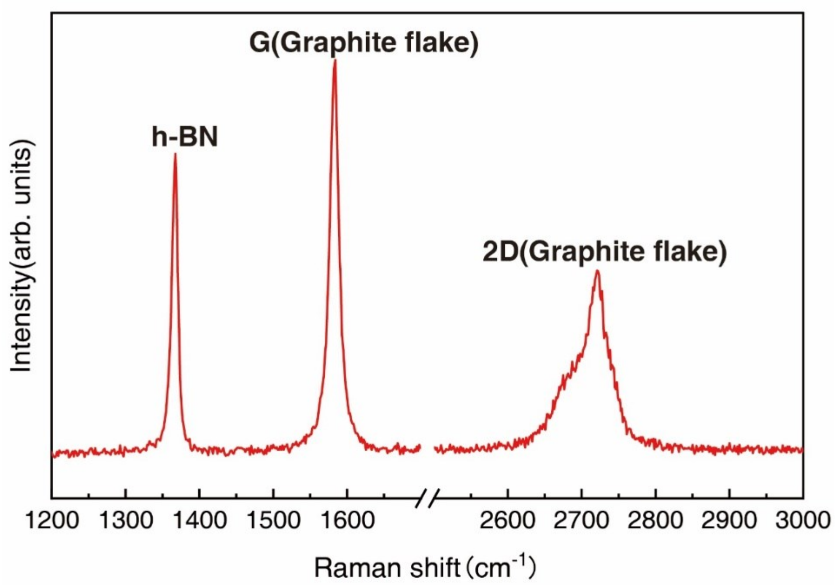

Supplementary Figure 3. Raman spectra of h-BN and Graphite flake. Three peaks at 1375 cm^{-1}, 1580 cm^{-1} and 2720 cm^{-1} corresponded to the h-BN, G peak and 2D band of GF, respectively. The sharp peaks indicate that the materials are of high quality and have no major defects.

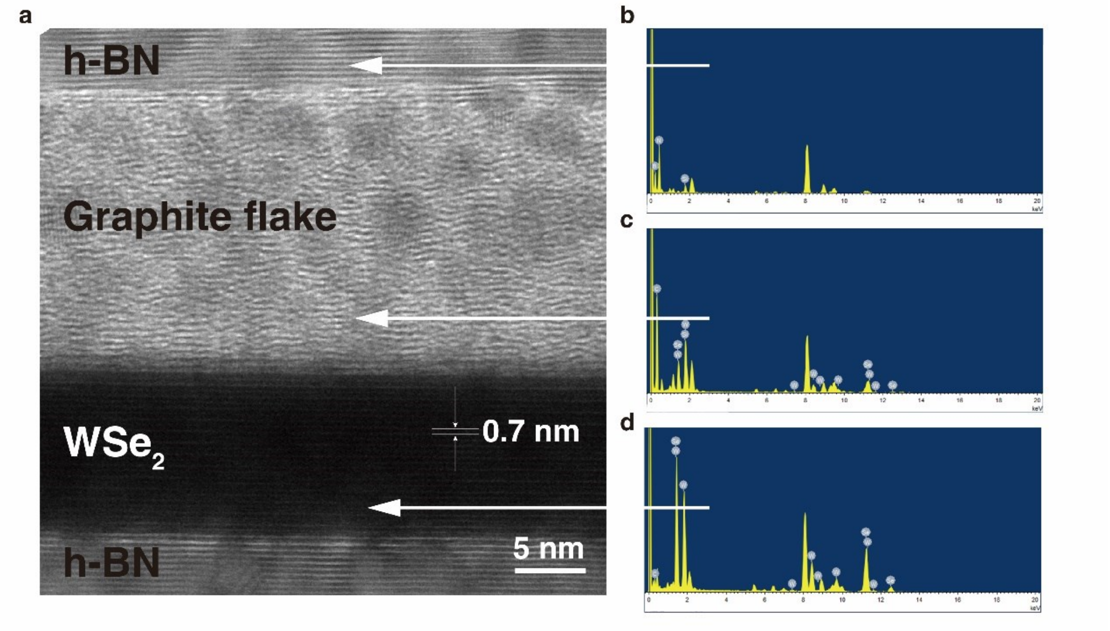

Supplementary Figure 4. High Resolution Transmission electron microscope (HRTEM) images of the stacked structure and elemental analysis results of

different areas. a, HRTEM image of the stacked structure. Clear lattice structure can be seen in the image, indicating excellent material quality. **b, c, d** are elemental analysis results of h-BN, GF, WSe_2, respectively. According to the results of element analysis, we can well determine the type of material in each area.

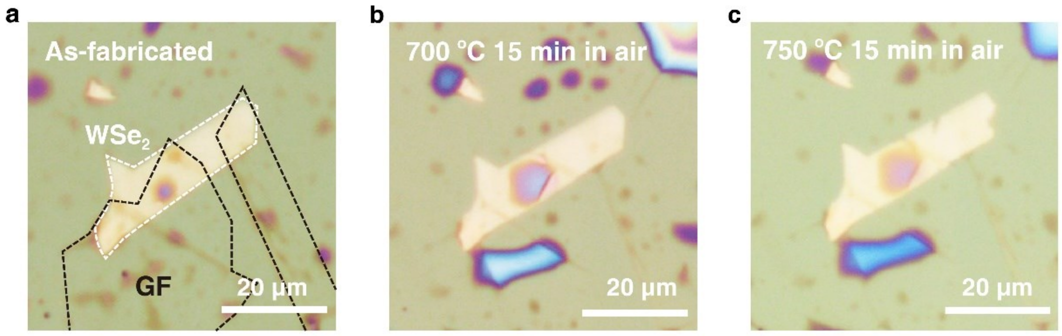

Supplementary Figure 5. Optical images of WSe_2 FET before and after 700 °C/750 °C burning. a, As-fabricated WSe_2 FET with h-BN encapsulation and GF electrodes. Dashed lines are used to indicate the outline of WSe_2 (white) and GF (black). **b,** WSe_2 FET after 700 °C heating for 15 min in air. No significant change of WSe_2 was observed. **c,** WSe_2 FET after 750 °C heating for 15 min in air. WSe_2 showed only very small defects at the edge.

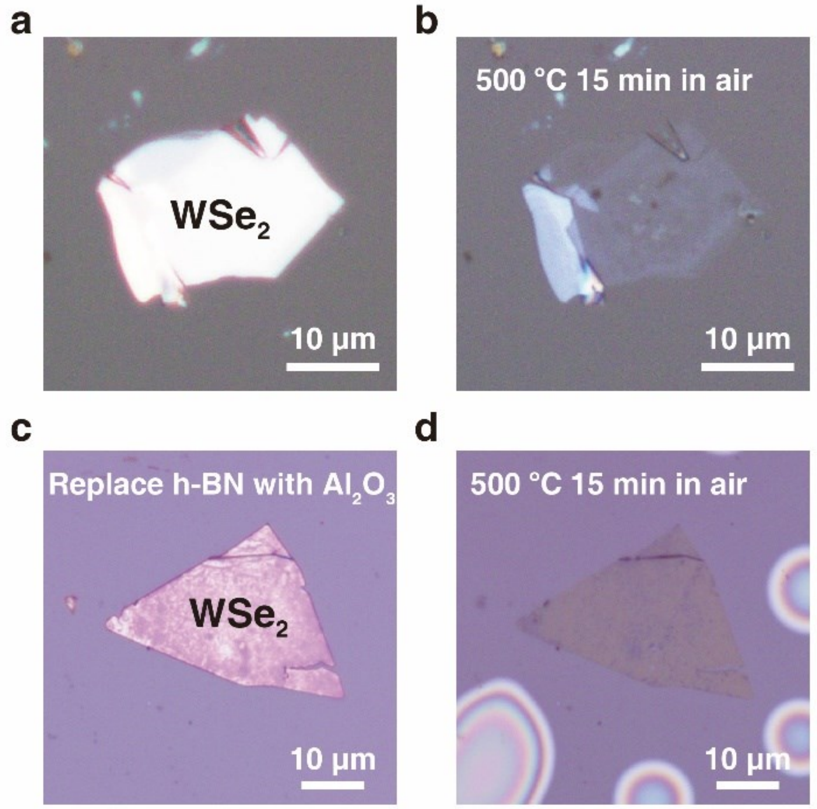

Supplementary Figure 6. Thermal stability of bare WSe_2 and WSe_2 covered with 100 nm Al_2O_3. a, Optical image of exfoliated WSe_2 on mica substrate. **b,** Optical image of the same WSe_2 after 500 °C burning for 15 min in air. The WSe_2 flake was strongly

oxidized and became nearly transparent inasmuch as WO_3 is transparent under visible light. **c,** Optical image of exfoliated WSe_2 on mica substrate covered with 100 nm Al_2O_3. **d,** Optical image of the WSe_2 covered with 100 nm Al_2O_3 after 500 °C burning for 15 min in air. The WSe_2 flakes were strongly oxidized and became nearly transparent.

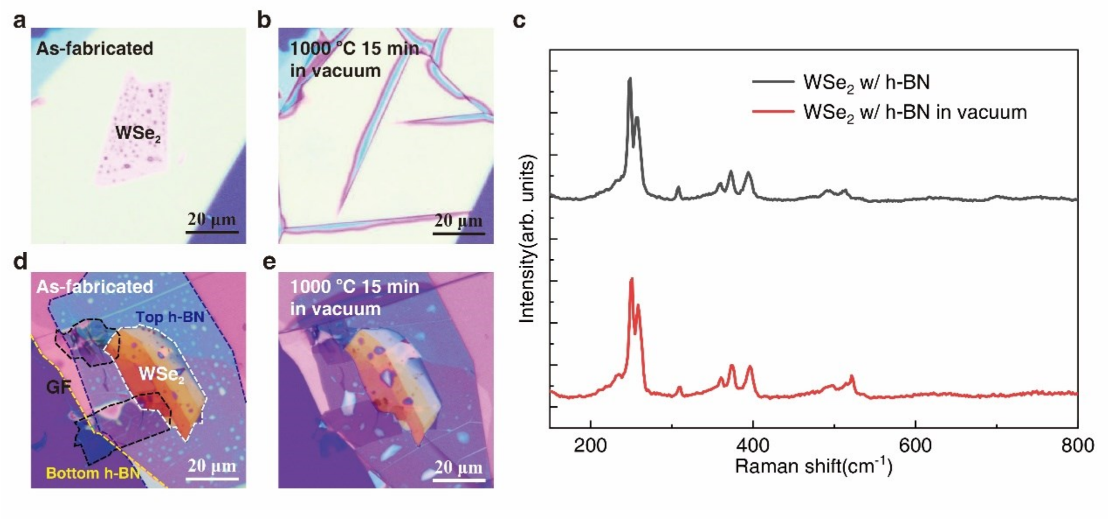

Supplementary Figure 7. WSe_2 FET before and after 1000 °C burning in vacuum. **a,** Bare WSe_2 before 1000 °C burning. **b,** The WSe_2 is vanished after 1000°C burning. **c,** Raman spectrum of WSe_2 after 1000°C burning in vacuum. **d,** WSe_2 FET before 1000°C burning. Dashed lines are used to indicate the outline of WSe_2 (white) and GF (black). **e,** WSe_2 FET after 1000°C burning in vacuum.

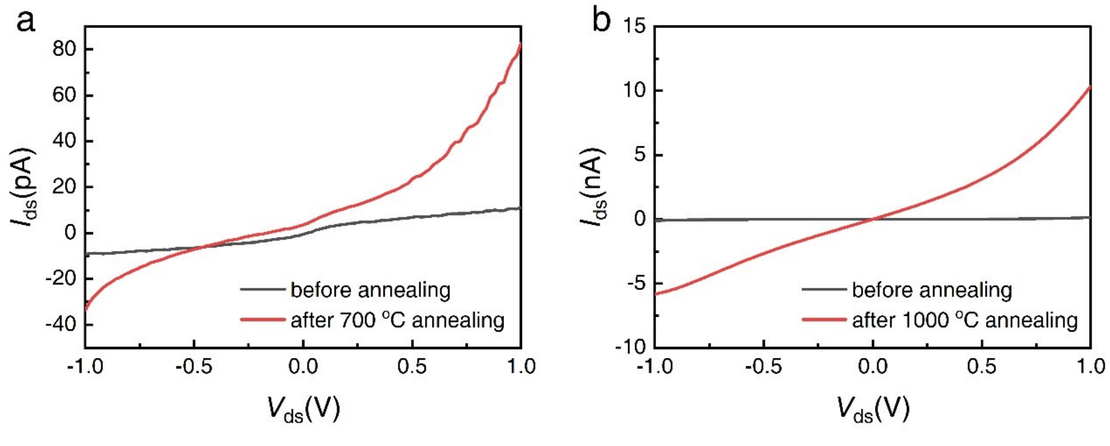

Supplementary Figure 8. Dark current before and after 700 °C in air and 1000 °C in vacuum annealing. In both situations, the current increased after high temperature annealing.

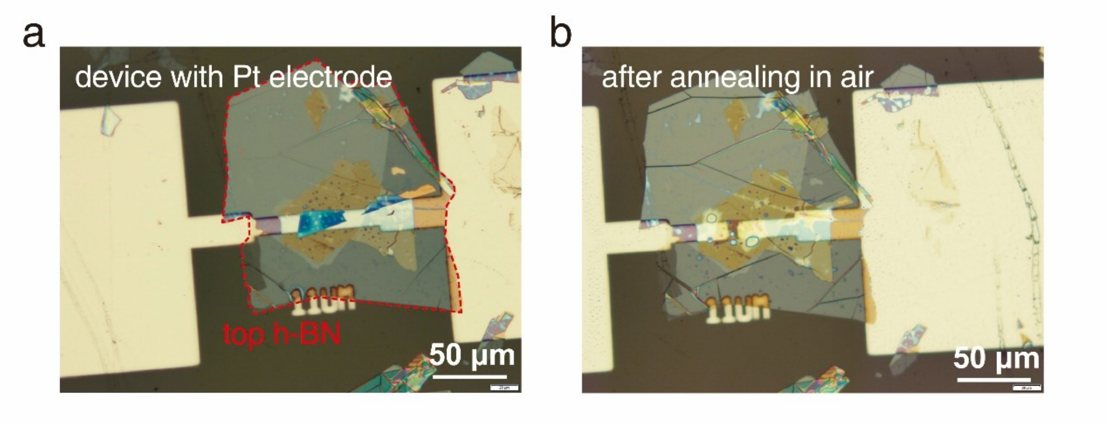

Supplementary Figure 9. Optical microscope image of a typical device with Pt electrodes before and after annealing. The channel material is strongly oxidized. Red dashed line is used to indicate the outline of top h-BN.

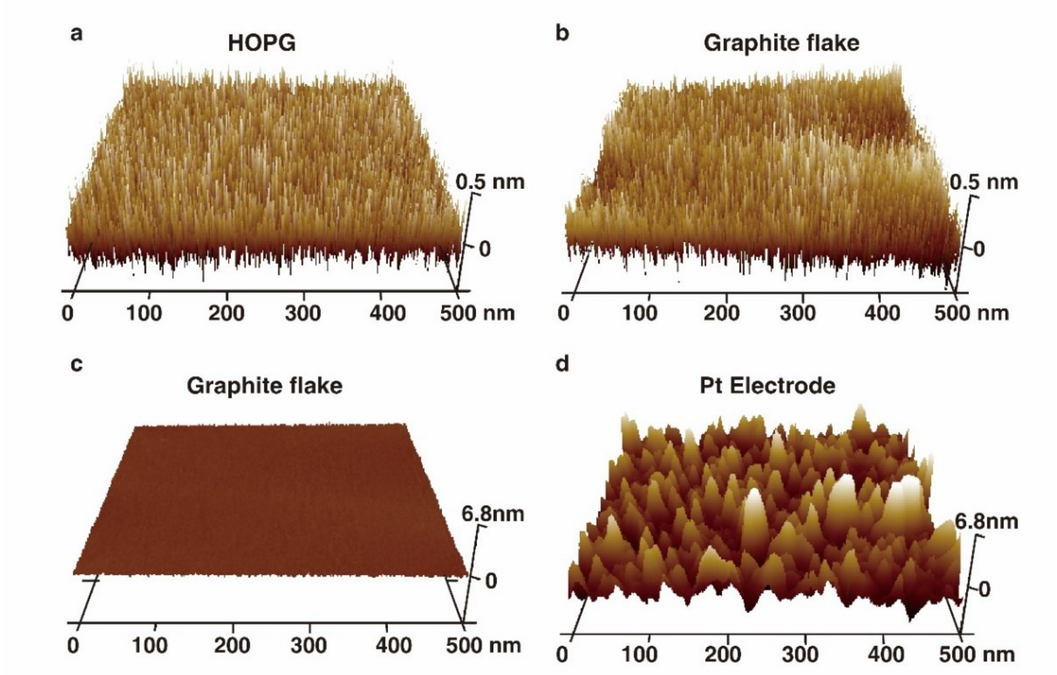

Supplementary Figure 10. Atomic force microscope (AFM) images of HOPG (highly oriented pyrolytic graphite), GF, and Pt electrode. (a) AFM image of HOPG. **(b), (c)** AFM image of exfoliated on h-BN in different Z direction scales. **(d)** AFM image of Pt electrode.

Note: HOPG is reported to have atomic flat surface. The height variation of HOPG surface is ~0.5 nm and exfoliated GF is ~0.6 nm, indicating that exfoliated GF has a surface flatness close to that of HOPG. The height variation of Pt electrode is ~6.8 nm, much larger than that of GF electrode (~0.6 nm). Instrument noise affected the measurement by approximately 0.4 nm, indicating that the height variation of GF was mainly limited by instrument noise. The result demonstrated that exfoliated GF had a much flatter surface than Pt electrodes. Consequently, good encapsulation can be formed between h-BN and GF.

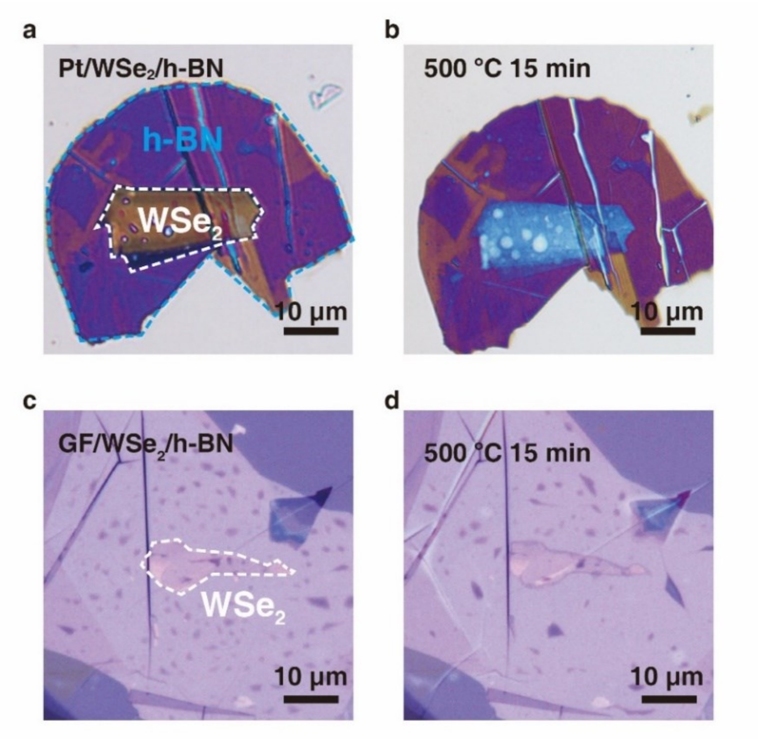

Supplementary Figure 11. Comparison of GF and Pt as bottom encapsulation layer. a, Optical images of Pt/WSe_2/h-BN structure using 30 nm thick Pt as bottom layer. Dashed lines are used to indicate the outline of WSe_2 (white) and top h-BN (blue). **b,** Optical images of Pt/WSe_2/h-BN structure after burning 15 min at 500 °C. **c,** Optical images of GF/WSe_2/h-BN structure using exfoliated GF as bottom layer. White dashed line is used to indicate the outline of WSe_2. **d,** Optical images of GF/WSe_2/h-BN structure after burning 15 min at 500 °C.

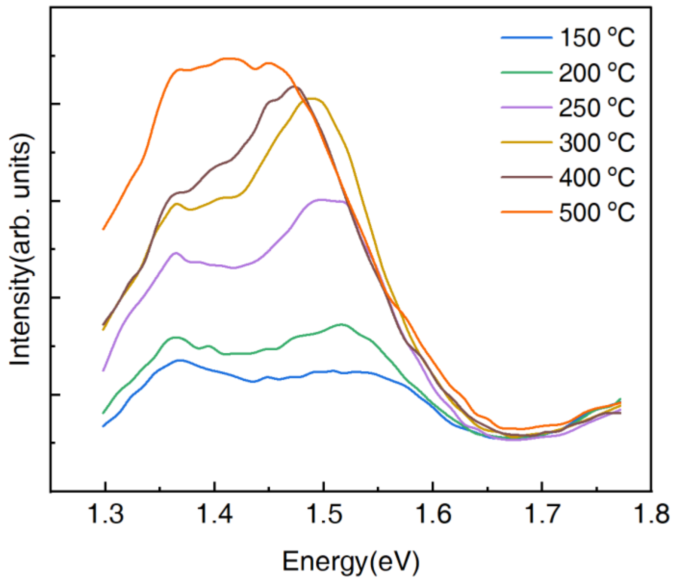

Supplementary Figure 12. Photoluminescence spectroscopy image of WSe_2 in heat resistant structure from 150 °C to 500 °C. The leftward shift of the PL peak with increasing temperature indicated a decreasing energy bandgap of WSe_2.

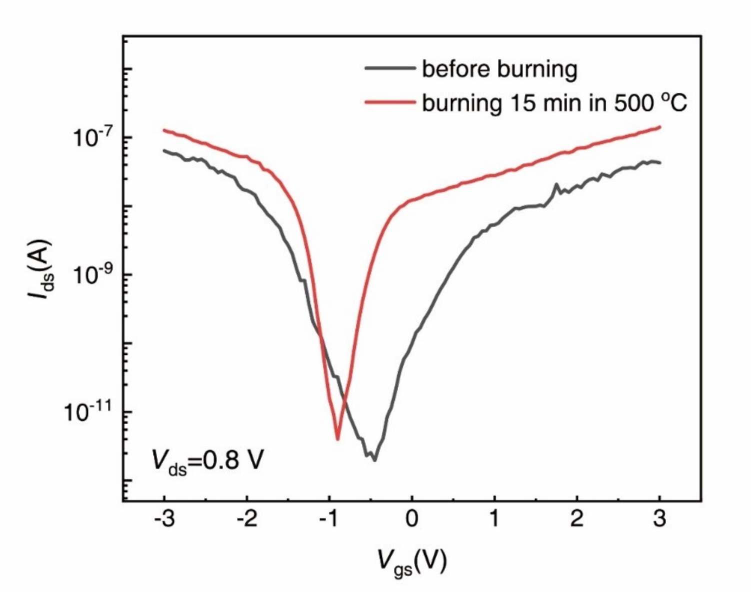

Supplementary Figure 13. I_{ds}-V_{gs} curves (V_{ds}=0.8V) measured at room temperature before and after burning 15 min at 500 °C in air.

Note: Both p-type and n-type currents were enhanced after burning at 500 °C in air, which may be due to improved contact by high temperature heating. The results further proved that heating at 500 °C will not cause significant damage to our devices, even improving the performance of the device as a result.

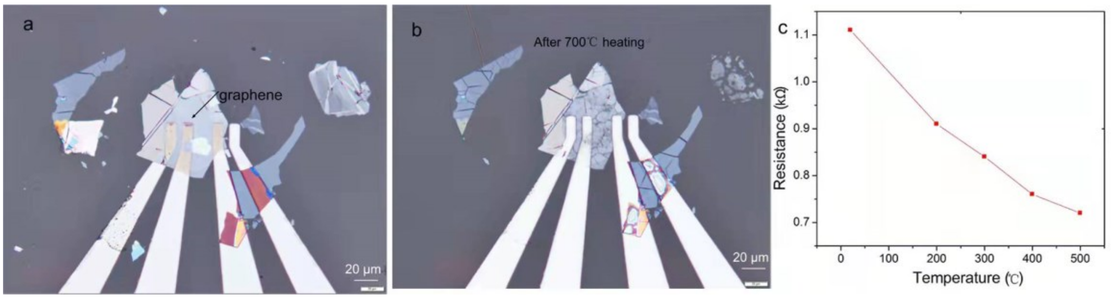

Supplementary Figure 14. Conducting properties of GF electrode at high-temperature. a, GF flake before heating. The resistance of the fresh GF flake is 1.2 kΩ. **b,** GF flake after 700°C heating for 15 min in air. The bare GF without h-BN protection is still in good shape with smaller resistance of 0.6 kΩ at room temperature. **c,** The resistance of a typical GF flake reduces from 1.1 kΩ to 0.7 kΩ as temperature varied from 20°C to 500°C.

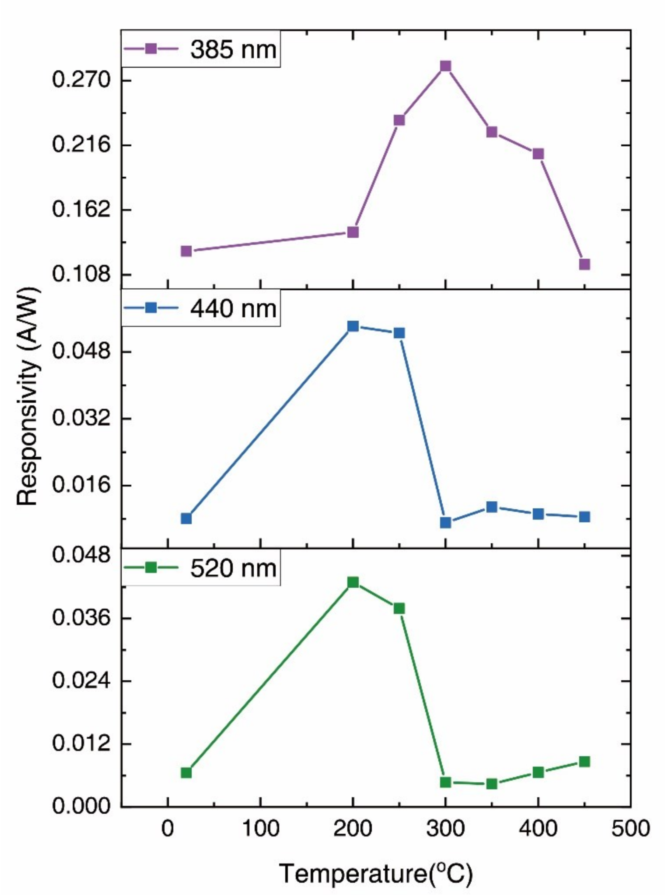

Supplementary Figure 15. Photo responsivity of WSe_2 device with mica encapsulation under 385 nm (purple), 440 nm (blue), and 520 nm (green) illumination at different temperatures (V_{ds}=0.4 V, V_g=0 V).

Note: Under the illumination of three different lights, all the responsivity increased and then decreased with increasing temperature from 20 °C to 450 °C in air, but remains positive. The device with top mica exhibited positive photoconductivity in 400 °C, indicating h-BN encapsulation contributed to the novel negative photoconductivity phenomenon in 400 °C.

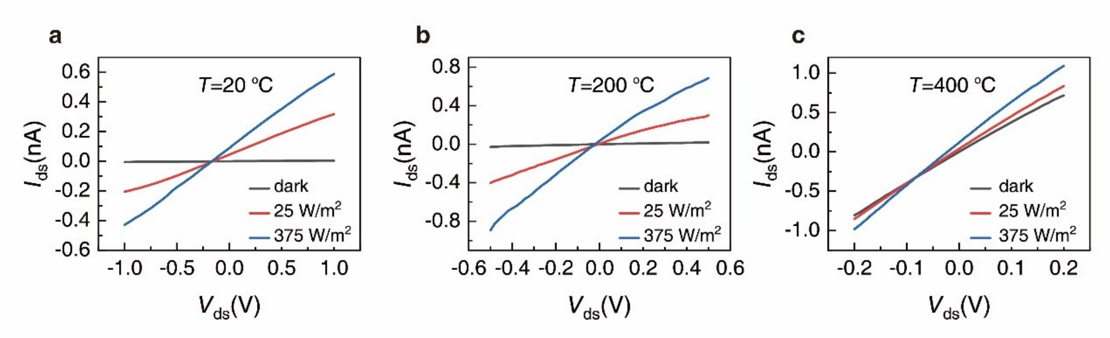

Supplementary Figure 16. The I_{ds}-V_{ds} curves of bare WSe_2 with GF electrodes (without top h-BN) under different light intensity and temperature. a, The I_{ds}-V_{ds} curves of bare WSe_2 with GF electrodes under white light illumination at 20 °C. **b,** The I_{ds}-V_{ds} curves of bare WSe_2 with GF electrodes under white light illumination at 200 °C.

c, The I_{ds}-V_{ds} curves of bare WSe_2 with GF electrodes under white light illumination at 400 °C.

Note: At three different temperatures, positive photoconductive effect was observed as illumination power increased, indicating that bare WSe_2 with GF electrodes (without top h-BN) showed positive photoconductive effect under illumination. The contribution of WSe_2 and WSe_2/GF electrode contact to the novel photoconductivity phenomenon in 400 °C have been excluded.

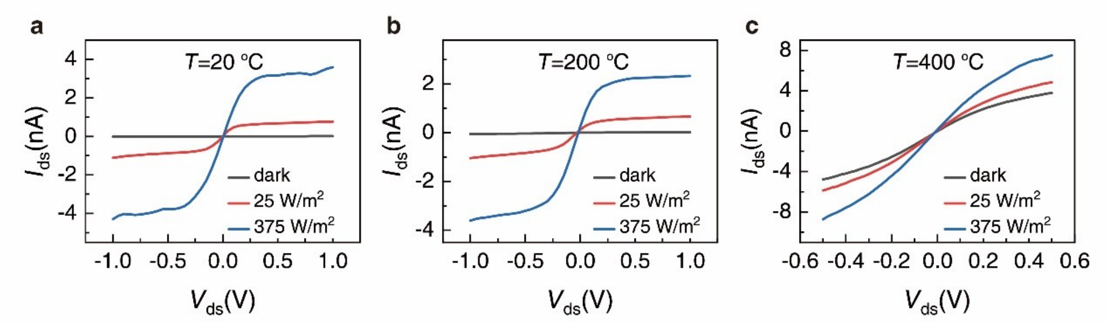

Supplementary Figure 17. The I_{ds}-V_{ds} curves of bare WSe_2 with Pt electrodes (without top h-BN) under different light intensity and temperature. a, The I_{ds}-V_{ds} curves of bare WSe_2 with Pt electrodes under white light illumination at 20 °C. **b,** The I_{ds}-V_{ds} curves of bare WSe_2 with Pt electrodes under white light illumination at 200 °C. **c,** The I_{ds}-V_{ds} curves of bare WSe_2 with Pt electrodes under white light illumination at 400 °C.

Note: In three different temperature, positive photoconductive effect was observed as illumination power increased, indicating that contact between WSe_2 and Pt is not the cause of the negative photoconductivity phenomenon in 400 °C.

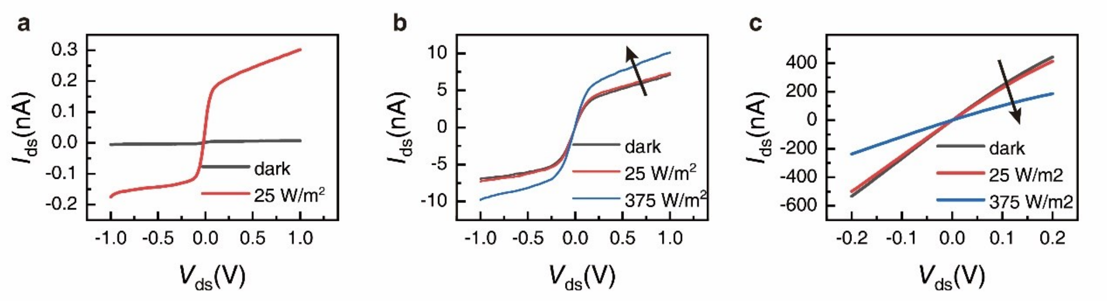

Supplementary Figure 18. The I_{ds}-V_{ds} curves of WSe_2 with Pt S/D electrodes and h-BN encapsulation (without top gate) under different light intensity and temperature. a, The I_{ds}-V_{ds} curves of bare WSe_2 with Pt electrodes under white light illumination at 20 °C. **b,** The I_{ds}-V_{ds} curves of bare WSe_2 with Pt electrodes under white light illumination at 200 °C. **c,** The I_{ds}-V_{ds} curves of bare WSe_2 with Pt electrodes under white light illumination at 400 °C.

Note: The current of WSe_2 device increased as light intensity increased from 25 W/m^2 to 375 W/m^2 at 20 °C and 400 °C. The current decreased with increasing light intensity, indicating negative photoconductivity.

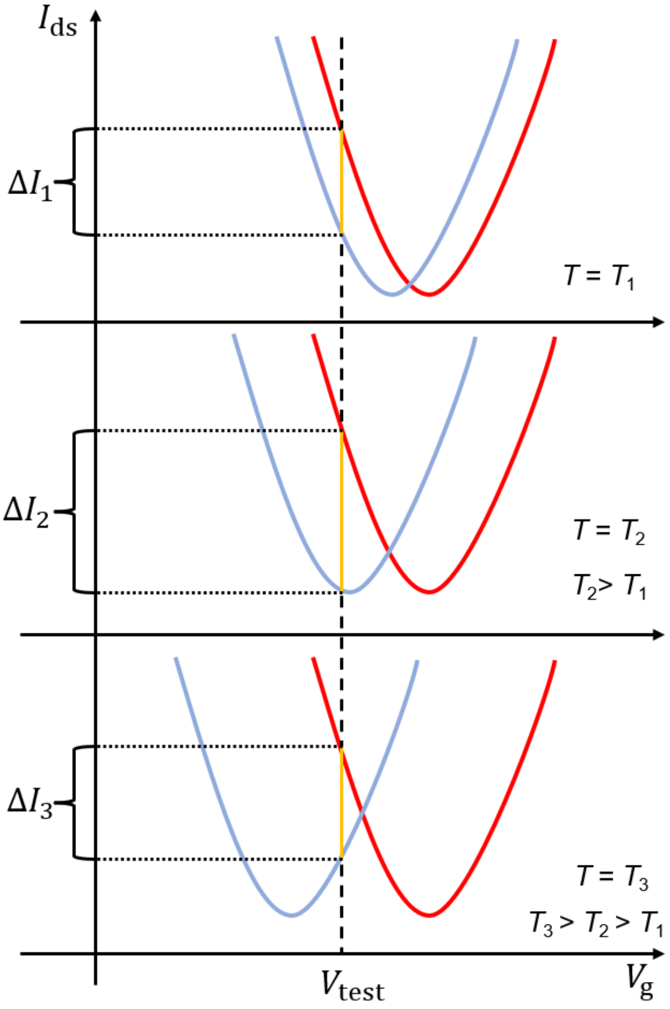

Supplementary Figure 19. Schematic of transfer curves at different temperatures. Since the I_{ds}-V_{gs} curve demonstrated ambipolar behavior, the photocurrent ΔI increases first and then decreases with the left shift of the curve when measured at the same V_g.

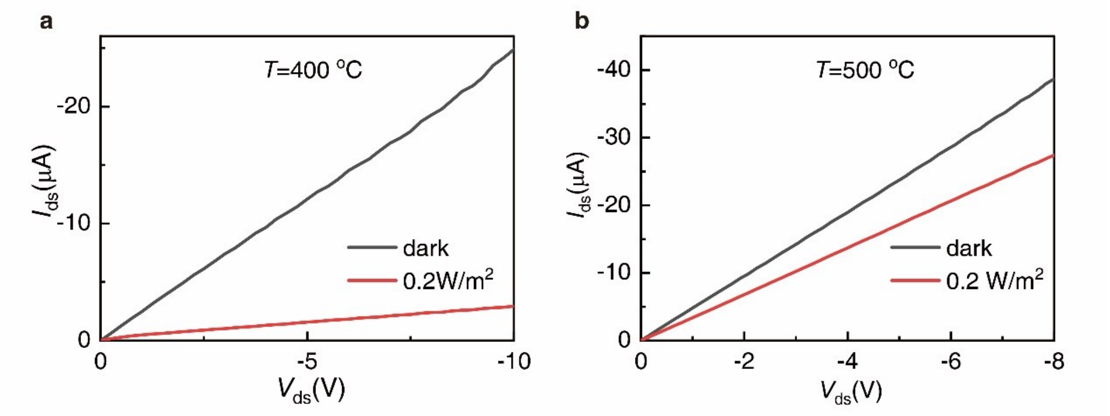

Supplementary Figure 20. Negative photoconductivity phenomenon at 400 °C and 500 °C under 0.2 W/m^2 365 nm light illumination. The photoresponsivity derived can reach 2.2×10^6 A/W and 1.1×10^6 A/W respectively.

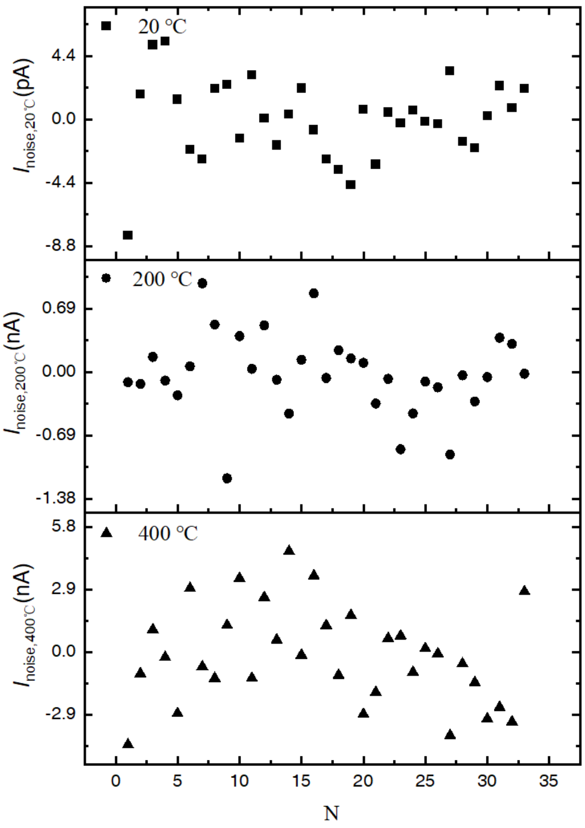

Supplementary Figure 21. Noise current sequence at different temperatures.

Note: The noise currents, signal currents and signal-to-noise ratio at different temperatures are listed in Supplementary Table 1. We use standard deviation of noise to represent I_{noise}. The I_{signal} values were all derived under 18 W/m^2 385 nm illumination. Compared to room temperature, the signal-to-noise ratio at 200 °C is significantly lower. This is because the high temperature greatly amplifies the noise while the PPC signal does not significantly increase. At 400°C, the device operated in NPC mode has a very high responsivity, resulting in an increased signal-to-noise ratio (152.9). The signal-to-noise ratio of our device at high temperature is sufficient to meet the needs of optical sensing.

Supplementary Table 1. Devices with different sizes of top h-BN

Size of top h-BN	Pt electrode devices	Graphite flake electrode devices
~5000 μm^2	oxidized	unchanged
~10000 μm^2	oxidized	unchanged
~15000 μm^2	oxidized	unchanged
~18000 μm^2	oxidized	unchanged
~22500 μm^2	oxidized	/

Supplementary Table 2. Signal-to-noise ratio of the device at different temperatures

Temperature(°C)	I_{noise}(A)	I_{signal}(A)	SNR	Device status
20	2.74×10^{-12}	3.11×10^{-9}	1135	PPC
200	4.64×10^{-10}	1.31×10^{-8}	28.2	PPC
400	2.06×10^{-9}	3.15×10^{-7}	152.9	NPC

Supplementary Table 3. Comparation of representative photodetectors

Materials	Wavelength[nm]	Temperature[°C]	D*[jones]	Reference
WSe_2(CVD)	500-900	RT	1×10^{14}	[1]
MoS_2	532	200	1×10^{10}	[2]
MoS_2	550-800	RT	7.7×10^{11}	[3]
WSe_2	532	RT	1.1×10^{12}	[4]
GaN	440	527	4×10^{8}	[5]
Ga_2O_3	270	RT	1×10^{12}	[6]
$Al_{0.4}Ga_{0.6}N$	280	127	2.4×10^{13}	[7]
SiC nanowire	254	RT	7.2×10^{10}	[8]
Si	1060	RT	1×10^{10}	[9]
Si	950	RT	1.5×10^{14}	[10]
WSe_2	365	400	1.6×10^{13}	This work
WSe_2	365	500	2.63×10^{12}	This work

Supplementary Reference:

1. Zhang, W. et al. Role of metal contacts in high-performance phototransistors based on WSe_2 monolayers. *ACS nano* **8**, 8653-8661 (2014).
2. Tsai, D. S. et al. Few-layer MoS_2 with high broadband photogain and fast optical switching for use in harsh environments. *ACS Nano* **7**, 3905-3911 (2013).
3. Kufer, D., & Konstantatos, G. Highly sensitive, encapsulated MoS_2 photodetector with gate controllable gain and speed. *Nano Lett.* **15**, 7307-7313 (2015).
4. Ghosh, S. et al. Enhanced responsivity and detectivity of fast WSe_2 phototransistor using electrostatically tunable in-plane lateral p-n homojunction. *Nat. Commun.* **12**, 1-9 (2021).
5. Madhusoodhanan, S. et al. High-Temperature Analysis of GaN-Based MQW Photodetector for Optical Galvanic Isolations in High-Density Integrated Power Modules. *IEEE Trans. Emerg. Sel. Topics Power Electron.* **9**, 3877-3882 (2020).
6. Tak, B. R. et al. Wearable gallium oxide solar-blind photodetectors on muscovite mica having ultrahigh photoresponsivity and detectivity with added high-temperature functionalities. *ACS Appl. Electron. Mater.* **1**, 2463-2470 (2019).
7. Kaushik, S. et al. Localized surface plasmon resonance-enhanced solar-blind $Al_{0.4}Ga_{0.6}N$ MSM photodetectors exhibiting high-temperature robustness. *Nanotechnology* **33**, 145202 (2022).
8. Yildirim, M. A., & Teker, K. Self-powered fine-pattern flexible SiC single nanowire ultraviolet photodetector. *J. Alloys Compd.* **868**, 159255 (2021).
9. Wang, L. et al. Highly sensitive narrowband Si photodetector with peak response at around 1060 nm. *IEEE Trans. Electron Devices* **67**, 3211-3214, (2020).
10. Guo, X.et al. High-responsivity Si photodiodes at 1060 nm in standard CMOS technology. *IEEE Electron Device Lett.* **39**, 228-231 (2017).
