# Supplementary material for: High-temperature flexible WSe2 photodetectors with ultrahigh photoresponsivity
Source: Nat Commun. 2022 Jul 28;13:4372. doi: 10.1038/s41467-022-32062-0 (PMC9334605; doi:10.1038/s41467-022-32062-0)
Supplement: Supplementary file 3 — Description of Additional Supplementary Files [file 41467_2022_32062_MOESM3_ESM.docx]

**Description of Additional Supplementary Files**

File Name: Supplementary Movie 1

Description: Flexibility of WSe2 photodetectors after heating at 700 ℃ in air for 15 minutes.
